# Mycothiol biosynthesis is essential for ethionamide susceptibility in *Mycobacterium tuberculosis*

**DOI:** 10.1111/j.1365-2958.2008.06365.x

**Published:** 2008-07-21

**Authors:** Catherine Vilchèze, Yossef Av-Gay, Rodgoun Attarian, Zhen Liu, Manzour H Hazbón, Roberto Colangeli, Bing Chen, Weijun Liu, David Alland, James C Sacchettini, William R Jacobs

**Affiliations:** 1 Howard Hughes Medical Institute, Department of Microbiology and Immunology, Albert Einstein College of MedicineBronx, NY 10461, USA; 2 Division of Infectious Diseases, University of British ColumbiaVancouver, BC V5Z 3J5, Canada; 3 Department of Biochemistry and Biophysics, Texas A&M UniversityCollege Station, TX 77843, USA; 4 Division of Infectious Disease, Department of Medicine and the Ruy V. Lourenço Center for the Study of Emerging and Re-emerging Pathogens, New Jersey Medical School, University of Medicine and Dentistry of New JerseyNewark, NJ 07103, USA

## Abstract

Spontaneous mutants of *Mycobacterium tuberculosis* that were resistant to the anti-tuberculosis drugs ethionamide and isoniazid were isolated and found to map to *mshA*, a gene encoding the first enzyme involved in the biosynthesis of mycothiol, a major low-molecular-weight thiol in *M. tuberculosis*. Seven independent missense or frameshift mutations within *mshA* were identified and characterized. Precise null deletion mutations of the *mshA* gene were generated by specialized transduction in three different strains of *M. tuberculosis*. The *mshA* deletion mutants were defective in mycothiol biosynthesis, were only ethionamide-resistant and required catalase to grow. Biochemical studies suggested that the mechanism of ethionamide resistance in *mshA* mutants was likely due to a defect in ethionamide activation. *In vivo*, a mycothiol-deficient strain grew normally in immunodeficient mice, but was slightly defective for growth in immunocompetent mice. Mutations in *mshA* demonstrate the non-essentiality of mycothiol for growth *in vitro* and *in vivo*, and provide a novel mechanism of ethionamide resistance in *M. tuberculosis.*

## Introduction

The increase in drug resistance in *Mycobacterium tuberculosis* clinical isolates has impeded the full success of tuberculosis (TB) control. The WHO estimates that 4.3% of the newly and previously treated TB cases are multi-drug-resistant (MDR) meaning that these strains are resistant to at least the two best anti-TB drugs: isoniazid (INH) and rifampicin (Zignol *et al*., 2006). Alarmingly, there has been an emergence of *M. tuberculosis* strains resistant to four to seven TB drugs (termed XDR-TB for extensively drug-resistant TB) that have been associated with the rapid death of HIV-infected individuals (Gandhi *et al*., 2006; Shah *et al*., 2007). A more effective treatment for both MDR- and XDR-TB strains requires rapid detection and therefore understanding of all the mechanisms leading to drug resistance.

Isoniazid, the cornerstone of front-line TB treatment, shares a common target with the second-line TB drug ethionamide (ETH). Both INH and ETH are pro-drugs that require activation to form adducts with NAD to subsequently inhibit InhA, the NADH-dependent enoyl-ACP reductase (Dessen *et al*., 1995; Quemard *et al*., 1995) of the fatty acid biosynthesis type II system (Marrakchi *et al*., 2000). However, activation of INH and of ETH occurs through different pathways. INH is activated by the *katG*-encoded catalase peroxidase (Zhang *et al*., 1992; Wilming and Johnsson, 1999) to form the INH-NAD adduct (Rozwarski *et al*., 1998). ETH, on the other hand, is activated by the *ethA*-encoded mono-oxygenase (Baulard *et al*., 2000; DeBarber *et al*., 2000; Vannelli *et al*., 2002) to yield the ETH-NAD adduct (Wang *et al*., 2007). Mutations in either activator confer resistance to INH or ETH respectively (Piatek *et al*., 2000; Morlock *et al*., 2003; Ramaswamy *et al*., 2003; Hazbon *et al*., 2006). Co-resistance to INH and ETH can be mediated by mutations that alter the InhA target so as to prevent the INH-NAD or the ETH-NAD adduct from binding (Vilcheze *et al*., 2006), by mutations that cause InhA overexpression (Larsen *et al*., 2002; Vilcheze *et al*., 2006) or by mutations in *ndh* that increase the intracellular NADH concentration, thereby competitively inhibiting the binding of the INH-NAD and ETH-NAD adducts to InhA (Miesel *et al*., 1998; Vilcheze *et al*., 2005). While the majority of clinical isolates resistant to INH or ETH have been shown to map to the activator genes (*katG*, *ethA*) or the *inhA* target, current studies still show that up to 22% of the INH-resistant *M. tuberculosis* clinical isolates have no mutations in the genes known to be involved in INH or ETH resistance (Hazbon *et al*., 2006). In this study, to identify novel mutations conferring INH and ETH resistance, we isolated spontaneous mutants of *M. tuberculosis in vitro* and found that they map to *mshA*, a gene encoding a glycosyltransferase involved in mycothiol biosynthesis, suggesting that *mshA* was non-essential. Additional genetic and biochemical studies demonstrated that mycothiol biosynthesis is required for ETH susceptibility in *M. tuberculosis*. Furthermore, *in vivo* studies showed that mycothiol is not required for growth in mice.

## Results and discussion

### Spontaneous mutants of *M. tuberculosis*, co-resistant to INH and ETH, map to *mshA*

Numerous studies have demonstrated that there exist strains of *M. tuberculosis* that are resistant to INH and do not have mutations in the genes associated with INH resistance (*katG*, *inhA* structural gene and promoter, *ndh*) (Telenti *et al*., 1997; Piatek *et al*., 2000; Ramaswamy *et al*., 2003; Cardoso *et al*., 2004; Hazbon *et al*., 2006). To eliminate the majority of spontaneous mutants of *M. tuberculosis* that are singly resistant to INH and map to *katG*, we chose to isolate mutants that were co-resistant to INH and its structural analogue ETH. Samples of three independent *M. tuberculosis* H37Rv cultures were plated on media containing low concentrations of both INH and ETH [≤ 4-fold the minimum inhibitory concentration (MIC)]. Seven mutants were isolated at low frequencies (1–4 × 10^−8^). DNA sequence analysis of targeted genes in these seven strains revealed the absence of mutations in the genes known to mediate co-resistance to INH and ETH, namely *inhA* (the gene or its promoter region) and *ndh*. This analysis provided the evidence that these strains possessed mutations that conferred INH and ETH resistance and had not been previously identified in *M. tuberculosis*. The mutants were transformed with a cosmid genomic library of the drug-susceptible *M. tuberculosis* parent. The frequency of transformation was extremely low for most of the mutants (less than 100 transformants per transformation), and only one mutant, mc^2^4936, which had the lowest level of INH resistance, yielded more than 1000 transformants. The cosmid transformants were screened for restoration of INH and ETH susceptibility. One potential complementing cosmid was isolated, sequenced and shown to contain the *mshA* gene, a gene characterized as mediating the first step in the biosynthesis of mycothiol (Newton *et al*., 2003; 2006), a key thiol in the family of *Actinomycetes* bacteria (Newton *et al*., 1996). A link between mycothiol biosynthesis and resistance to INH and ETH had been previously established in *Mycobacterium smegmatis* when transposon mutants in *mshA* were found to be resistant to INH (more than 25-fold) and ETH (sixfold) (Newton *et al*., 1999; 2003; Rawat *et al*., 2003). Subsequent sequence analysis of mc^2^4936 and the other mutants showed that all the *M. tuberculosis* H37Rv mutants had missense, nonsense or frameshift mutations in *mshA* (Table 1). The *mshA* mutants had various levels of resistance to INH (2- to 16-fold) and ETH (four- to eightfold) (Table 2). This is the first report that *mshA* mutations confer co-resistance to INH and ETH in *M. tuberculosis*.

**Table 1:** *M. tuberculosis* strains used in this study.

		*mshA* allele characterization			
				
Strain	Genotype	Nucleotide	Amino acid	Mutant generation	Source
H37Rv	*mshA1*	–	–		Trudeau Institute
mc^2^4931	*mshA3*	c382t	Stop codon AA128	Spontaneous mutant of H37Rv	This work
mc^2^4932	*mshA4*	c817t	R273C	Spontaneous mutant of H37Rv	This work
mc^2^4933	*mshA5*	g895t	G299C	Spontaneous mutant of H37Rv	This work
mc^2^4934	*mshA6*	c991t	Stop codon AA331	Spontaneous mutant of H37Rv	This work
mc^2^4935	*mshA7*	g1067a	G356D	Spontaneous mutant of H37Rv	This work
mc^2^4936	*mshA8*	a1082c	E361A	Spontaneous mutant of H37Rv	This work
mc^2^4937	*mshA9*	a1242del	Frameshift	Spontaneous mutant of H37Rv	This work
mc^2^4938	*mshA10*	Δ*mshA*		Specialized transduction with phAE222	This work
CDC1551	*mshA1*	–	–		CSU
mc^2^4939	*mshA10*	Δ*mshA*		Specialized transduction with phAE222	This work
Erdman	*mshA2*	a332g	N111S		Trudeau Institute
mc^2^4942	*mshA10*	Δ*mshA*		Specialized transduction with phAE222	This work

CSU, Colorado State University.

**Table 2:** INH and ETH minimum inhibitory concentrations (MICs).

	MIC (mg l^−1^)		MIC (mg l^−1^) pMV361::*mshA*	
		
Strain	INH	ETH	INH	ETH
H37Rv	0.06	2.5	0.06	2.5
mc^2^4931	0.6	20	0.06	2.5
mc^2^4932	0.4	10	0.06	2.5
mc^2^4933	0.6	20	0.12	2.5
mc^2^4934	1	10	0.5	2.5
mc^2^4935	1	10	0.06	2.5
mc^2^4936	0.12	20	0.06	2.5
mc^2^4937	0.5	20	0.12	2.5
mc^2^4938	0.06	> 20	0.06	2.5
CDC1551	0.06	2.5		
mc^2^4939	0.12	> 20	0.06	2.5
Erdman	0.06	2.5		
mc^2^4942	0.06	15	0.06	2.5

### The *mshA* mutants of *M. tuberculosis* are defective in the synthesis of mycothiol

**Fig. 1:**
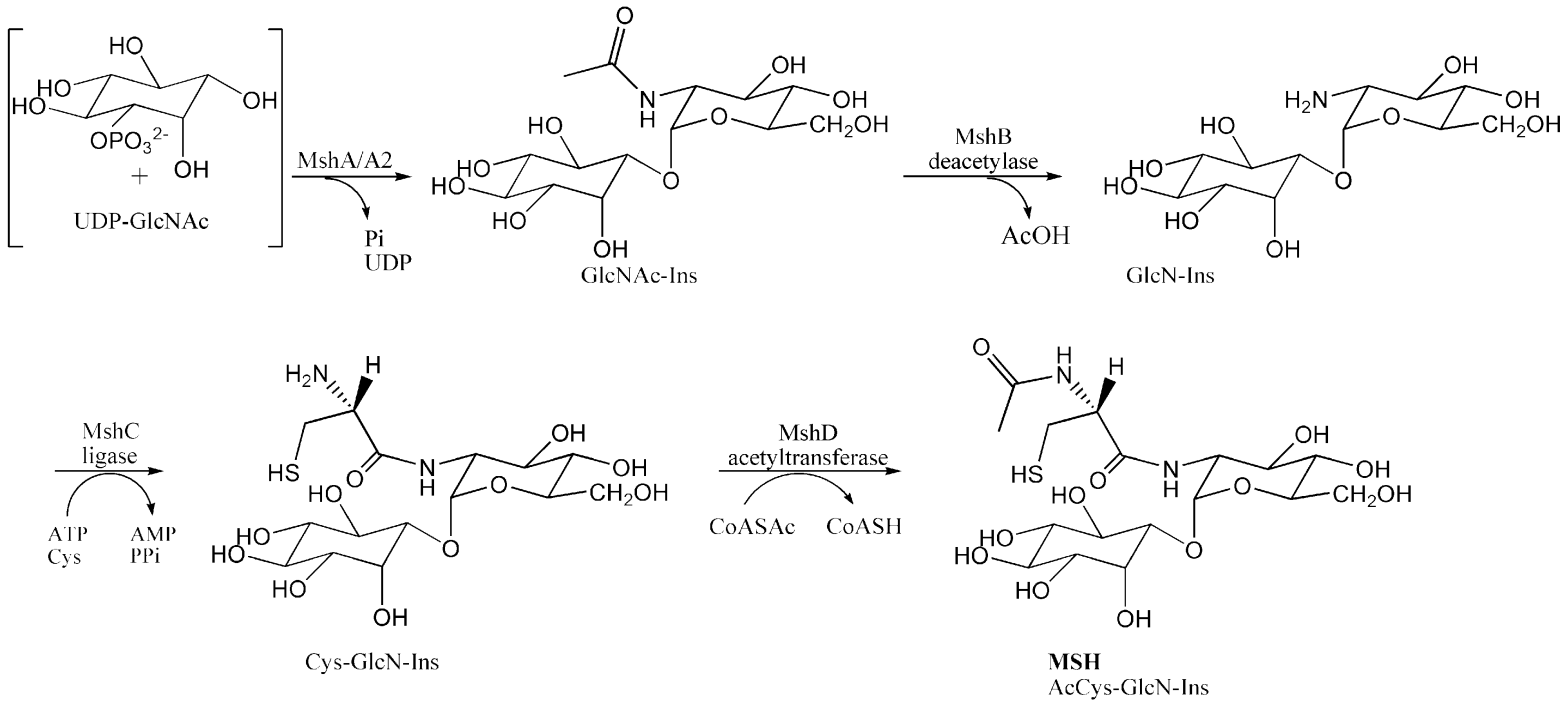
Mycothiol biosynthesis pathway. The first step in the biosynthesis of mycothiol (MSH) is the coupling of UDP-*N*-acetyl glucosamine and inositol-1-phosphate followed by dephosphorylation, carried out by the glycosyltransferase MshA and the phosphatase MshA2 respectively. The deacetylase MshB removes the acetyl group on the glucosamine which allows for the addition of a cysteine group on the free amino group by the ligase MshC. The last step is the acetylation of the cysteine amino group by the acetyltransferase MshD.

**Fig. 2:**
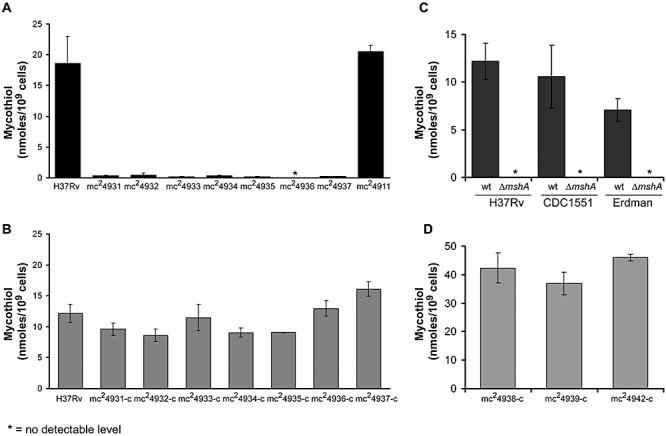
Mycothiol contents in *M. tuberculosis mshA* mutants and complemented strains. The strains were grown to stationary phase. Mycothiol contents were measured in triplicate for the *M. tuberculosis mshA* point mutants (A) and their complemented strains (B) as well as for the *mshA* null mutants (C) and their complemented strains (D) as described in *Experimental procedures*. c = pMV361::*mshA*.

The glycosyltransferase MshA is the first step in mycothiol biosynthesis that leads to the formation of *N-*acetyl-glucosamine inositol (Newton and Fahey, 2002; Newton *et al.*, 2006). The biosynthesis of mycothiol requires five enzymes to form *N*-acetyl-cysteine glucosamine inositol or mycothiol from inositol-1-phosphate and UDP-*N*-acetyl-glucosamine: the glycosyltransferase MshA, the phosphatase MshA2, the deacetylase MshB, the cysteine ligase MshC and the acetyltransferase MshD (Fig. 1). To analyse the effects of the diverse mutations in *mshA* on the biosynthesis of mycothiol, the levels of mycothiol were measured in all the mutants using fluorescent high-performance liquid chromatography (HPLC) assay (Newton *et al*., 2000a). We found a dramatic reduction (83% to undetectable levels) in the concentration of mycothiol compared with wild type (Fig. 2A). As a control, we also measured the mycothiol level in an INH- and ETH-resistant *M. tuberculosis inhA* mutant, mc^2^4911 (Vilcheze *et al*., 2006), and found that this mutant had a concentration of mycothiol similar to that in wild type. Complementation of the mutants with pMV361::*mshA*, an integrative plasmid containing only the *mshA* gene of *M. tuberculosis* driven by the *hsp60* promoter, restored mycothiol biosynthesis in all the mutants (Fig. 2B). This confirms that the defect in mycothiol biosynthesis was due to the mutations in *mshA.* Although mycothiol has been suggested to be essential for the growth of *M. tuberculosis* (Sareen *et al*., 2003), our data show that *M. tuberculosis* strains that do not produce mycothiol are viable.

### Comparison of the MshA structures of *M. tuberculosis* and *Corynebacterium glutamicum* establishes a rationale for the inactivation of MshA in the mutants

**Fig. 3:**
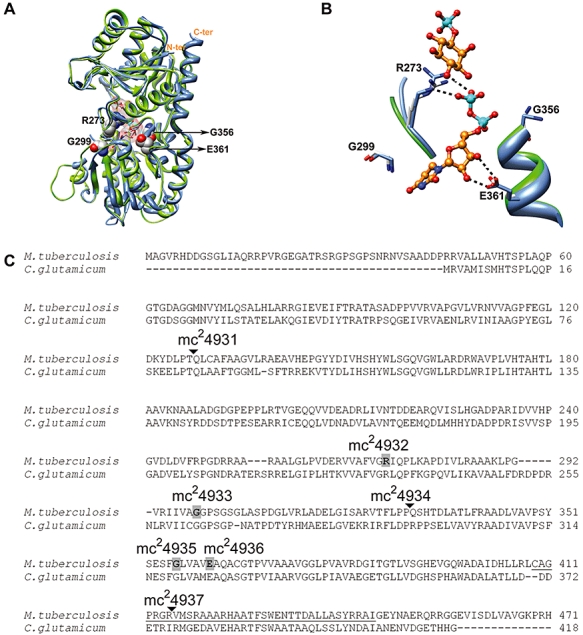
A. Ribbon representation of the superimposed *M. tuberculosis* MshA model (green) and CgMshA structure (blue) complexed with UDP/inositol-phosphate. Both UDP and inositol-phosphate are shown as stick with transparent surface. Four amino acids, whose mutation led to *M. tuberculosis* MshA inactivation, are represented as CPK structures and labelled accordingly. B. Active-site architecture of the superimposed MshA structures shown in (A). UDP and inositol-phosphate are shown as ball and stick. The four amino acid mutations R273, G299, G356 and E361, highlighted in (A), are shown as stick in white scheme, and the conserved residues in CgMshA are in white scheme, and the conserved residues in CgMshA are in blue scheme. Hydrogen bonds between the side-chain amines of Arg273 and the β-phosphate of UDP, as well as those between the side-chain carboxylate of Glu361 and the ribosyl hydroxyl groups of UDP are shown as black dotted lines. The residue numbers are for *M. tuberculosis* MshA. C. Alignment of the *M. tuberculosis* and *C. glutamicum mshA* sequences. The mutations identified in the *M. tuberculosis mshA* mutants are indicated. The four amino acid changes are in bold. The stop codons or frameshift are pointed by arrows. The residues in the α-helix crossing from C-terminal to N-terminal domain are underlined.

Given the sequence identity (45.9%) between *M. tuberculosis* MshA and *Corynebacterium glutamicum* MshA (CgMshA) whose structure was recently determined (Vetting *et al*., 2008), the monomeric homology model of *M. tuberculosis* MshA was created using CPHmodels 2.0 with the UDP-complexed CgMshA (PDB code 3C4Q) as template (Lund *et al*., 2002). Superimposition of the model of *M. tuberculosis* MshA (consisting of Arg46–Ile445) and the chain B from UDP/inositol-phosphate-bound CgMshA (PDB code 3C4V) yields an RMSD of 0.65 Å, indicating a high homology between each other (Fig. 3A).

Four of the *mshA* mutants have single amino acid mutation (Table 1). These four amino acids (Arg273, Gly299, Gly356 and Glu361) are conserved in CgMshA (as Arg231, Gly263, Gly319 and Glu324) (Fig. 3C). Each of these amino acids plays an important role in either the substrate binding or the domain interaction (Fig. 3B). The side-chain amines of Arg273 interact with the β-phosphate of UDP via hydrogen bonding. This arginine is also one of the major determinants of the orientation of the inositol-phosphate as its side-chain lies against the face of inositol. Gly299 is not in the vicinity of the active site, but should be important for the protein stability as the next residue, Gly300, forms the only interdomain hydrogen bond with Gly61 (Fig. 3A). Although not directly seen in the model, Gly356 was proposed to be involved in the binding of the *N*-acetyl-glucosamine moiety which shall be transferred from UDP to inositol (Vetting *et al*., 2008). In mc^2^4936, the Glu361Ala mutation removes the side-chain carboxylate that forms hydrogen bonds with the 2′- and 3′-hydroxyls from the ribose moiety of UDP, which could result in the inactivation of MshA.

The other *mshA* mutants had either nonsense or frameshift mutations (Fig. 3C). In mc^2^4931 and mc^2^4934, the nonsense mutations caused the loss of active-site elements. In mc^2^4937, the truncation of the protein was close to the C-terminus and the active site was unlikely to be affected. Herein the inactivation of MshA could be explained by the protein's characteristic folding. Based on the homology model of MshA, each monomer is composed of N-terminal and C-terminal domains. Towards the end of C-terminus, a large α-helix spanning Cys409 to Ile445 crosses back to the N-terminal, which is likely to stabilize the overall folding of the protein. Therefore the mutations within this α-helix, such as in mc^2^4937, would detrimentally affect the conformation of MshA leading to its inactivation.

### Co-resistance to INH and ETH is not mediated by *inhA* overexpression nor by increased NADH/NAD^*+*^ ratios in *mshA* mutants of *M. tuberculosis*

**Fig. 4:**
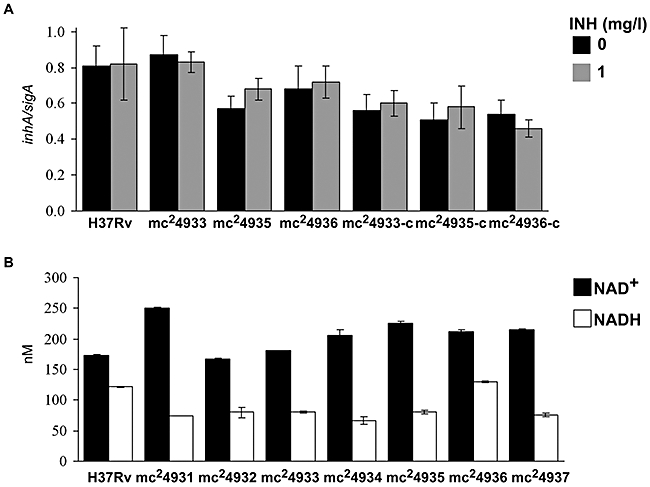
Possible mechanisms of INH and ETH resistance in *mshA* mutants. A. Three *mshA* point mutants and their complemented strains, as well as wild type, were either treated or not treated with INH (1 mg l^−1^) for 4 h before the *inhA* mRNA levels were measured. *inhA* levels were normalized to *sigA* expression. The experiment was performed in triplicate. B. NADH and NAD^+^ concentrations in *mshA* point mutants. The strains were grown to log phase. NADH and NAD^+^ were extracted and measured spectrophotometrically, as described in *Experimental procedures*. c = pMV361::*mshA*.

Although co-resistance to INH and ETH had been previously identified in *M. smegmatis mshA* transposon mutants (Rawat *et al*., 2003), no mechanism of resistance had been identified to link mycothiol biosynthesis with INH and ETH resistance (Koledin *et al*., 2002; Rawat *et al*., 2003). Previously, co-resistance to INH and ETH has been shown to be conferred by three different mechanisms: (i) structural mutations in the *inhA* target (Banerjee *et al*., 1994; Vilcheze *et al*., 2006), (ii) *inhA* target overexpression (Larsen *et al*., 2002; Vilcheze *et al*., 2006), or (iii) increased NADH/NAD^+^ ratios resulting in higher concentration of NADH, which competitively inhibits the binding of the INH-NAD or ETH-NAD adduct to InhA (Miesel *et al*., 1998; Vilcheze *et al*., 2005). We reasoned that it was highly unlikely that the gene product of *mshA* directly interacted with InhA. However, it was possible that mutations in *mshA* caused overexpression of *inhA* or altered the NADH/NAD^+^ ratios inside the *M. tuberculosis* cells. To test these possibilities, we first measured the *inhA* mRNA levels in three *mshA* mutants and their complemented strains using a molecular beacon reverse transcription polymerase chain reaction (RT-PCR) assay (Larsen *et al*., 2002). In contrast to the P-15 *mabA inhA* mutation, which has been shown to confer 10-fold overexpression of the *inhA* mRNA (Vilcheze *et al*., 2006), all three of these *mshA* mutants revealed no increase in *inhA* mRNA levels (Fig. 4A) and so resistance to ETH and INH was not due to InhA overexpression. We also measured the NAD^+^ and NADH concentrations in each of the mutants and found that the mutants had mostly lower NADH concentrations compared with wild type (Fig. 4B), demonstrating that the co-resistance to INH and ETH was not due to an increase in the NADH/NAD^+^ ratio. The sum of this work suggested that the *mshA* mutations must mediate a novel mechanism of resistance.

### Isolation of precise null deletions of *mshA* in various *M. tuberculosis* strains

Introduction of a wild-type copy of *mshA* restored ETH sensitivity (Table 2) and mycothiol content (Fig. 2B) in all the *mshA* mutants, but a subset of the mutants (3/7) did not regain INH susceptibility (Table 2). We reasoned that these strains must have acquired secondary mutations to compensate for the loss of mycothiol and that these mutations were also mediating the INH resistance. Although further studies will be needed to identify such mutations, we could hypothesize from these complementation studies that the loss of the *mshA* gene would mostly confer ETH resistance. To test this possibility, precise null mutants of *mshA* were generated in three reference strains of *M. tuberculosis* using specialized transduction (Bardarov *et al*., 2002) (Fig. S1, Table 1). The H37Rv, CDC1551 and the Erdman Δ*mshA* strains were resistant to ETH, but only the CDC1551 Δ*mshA* strain showed a twofold increase in INH MIC (Table 2). Previously, deletions in *mshB* (Buchmeier *et al*., 2003) and *mshD* (Buchmeier *et al*., 2006) had been isolated in *M. tuberculosis*, and only the *mshB* mutant was shown to be INH-resistant. Our data show that the main drug resistance phenotype of the *M. tuberculosis mshA* null mutant is resistance to ETH.

All three *M. tuberculosis mshA* deletion mutants failed to produce any detectable level of mycothiol (Fig. 2C). Complementation of the null mutants with pMV361::*mshA* restored ETH susceptibility (Table 2) and mycothiol production (Fig. 2D). To rule out the possibility that the lack of mycothiol might cause a compensatory phenotype, we measured total thiol concentrations in the cells and found that the null mutants' thiol concentrations were reduced by 53%, 77% and 82% in *M. tuberculosis* H37Rv, CDC1551 and Erdman respectively.

A previous study had suggested that the *mshA* gene was essential in *M. tuberculosis* (Buchmeier and Fahey, 2006). The discovery of frameshift mutations within the *mshA* gene, followed by our successful construction of null *mshA* deletions in three independent *M. tuberculosis* strains, demonstrates that *mshA* is not an essential gene in *M. tuberculosis.* Although both studies attempted to generate null *mshA* mutants in *M. tuberculosis* using specialized transduction, it would be difficult to extrapolate why one was successful and the other one was not. We can only note that the *M. tuberculosis mshA* deletion strains were obtained after a very long incubation at 37°C (8 weeks). Furthermore, this study also confirms that mycothiol is the major thiol in *M. tuberculosis* as it represents more than 50% of the total thiol concentration in *M. tuberculosis*.

### The *mshA* mutants require catalase to grow

**Fig. 5:**
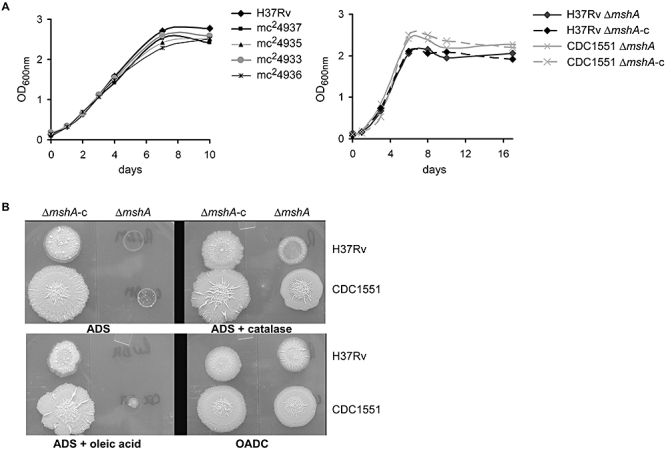
Growth of *M. tuberculosis mshA* mutants *in vitro*. A. Growth in liquid media (Middlebrook 7H9 supplemented with 0.2% glycerol, OADC and 0.05% tyloxapol) of four *mshA* point mutants (left) or two Δ*mshA* mutants and their complemented strains (right). B. Growth on solid media of H37Rv Δ*mshA* (top right), H37Rv Δ*mshA* pMV361::*mshA* (top left), CDC1551 Δ*mshA* (bottom right) and CDC1551 Δ*mshA* pMV361::*mshA* (bottom left) on Middlebrook 7H10 plates supplemented with 0.2% glycerol and ADS, ADS + beef liver catalase, ADS + oleic acid or OADC. c = pMV361::*mshA*.

As mycothiol has been suggested to be essential for the growth of *M. tuberculosis* (Sareen *et al*., 2003), we tested whether the *mshA* mutants had any growth defect *in vitro*. We observed no differences in growth rates in liquid media for the *mshA* point mutants and the null mutants (Fig. 5A). A previous study showed that an *M. tuberculosis* Δ*mshD* strain producing 1% of mycothiol compared with wild type required OADC (oleic acid-bovine albumin-dextrose-catalase-sodium chloride) to grow on plate (Buchmeier *et al*., 2006). We tested the *M. tuberculosis* Δ*mshA* mutants on Middlebrook 7H10 plates supplemented with glycerol and either OADC or ADS (bovine albumin-dextrose-sodium chloride). The mutants did not grow on the ADS plates but grew well on OADC plates. We then tested whether the mutants required either oleic acid and/or beef liver catalase to grow, as these are found in OADC supplement but not in ADS. Adding oleic acid to the ADS plate did not allow for growth of the mutants, but the addition of beef liver catalase was sufficient to restore growth on plates (Fig. 5B). The role of catalase in the OADC supplement is to eliminate toxic peroxides in the media. As mycothiol is involved in the detoxification of electrophiles, alkylating agents, antibiotics and oxidants (Rawat *et al*., 2007), it is not surprising that *M. tuberculosis* mutants producing no mycothiol require catalase for protection against toxic reactive oxygen intermediates.

### Mycolic acid biosynthesis is not inhibited by ETH treatment of *mshA* mutants

**Fig. 6:**
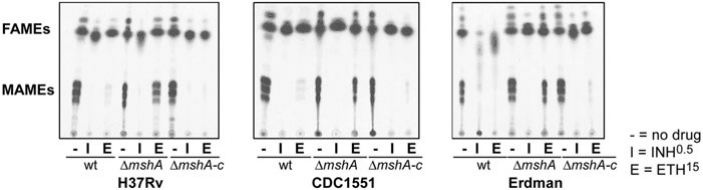
Fatty acid methyl ester (FAME) and mycolic acid methyl ester (MAME) analyses of Δ*mshA* mutants. *M. tuberculosis* wild-type strains, Δ*mshA* and complemented strains were treated with INH (0.5 mg l^−1^) or ETH (15 mg l^−1^) for 4 h, and then labelled with [1-^14^C]-acetate for 20 h. Fatty acids and mycolic acids were saponified, methylated, extracted and separated by thin-layer chromatography. ^14^C-labelled FAMEs and MAMEs were detected by autoradioragraphy after 36 h exposure at −80°C. c = pMV361::*mshA*.

The death of the tubercle bacillus following treatment with INH correlates with inhibition of the biosynthesis of the long-chain α-alkyl β-hydroxy fatty acids (up to 90 carbons in length) called mycolic acids, which are a major constituent of the mycobacterial cell wall (Winder and Collins, 1970; Takayama *et al*., 1972). ETH, based on its similarity to INH, has also been predicted and shown to inhibit mycolic acid biosynthesis (Winder *et al*., 1971; Quemard *et al*., 1992; Baulard *et al*., 2000). As mycothiol is not known to be involved in the FASII pathway, the resistance mediated by *mshA* could suggest that the lethal event occurs in some redox function. If so, it may be possible that INH and ETH treatment of *mshA* mutants does not confer resistance to mycolic acid inhibition by ETH or INH. Fatty acids were extracted from the wild-type *M. tuberculosis* strains, the Δ*mshA* mutants and the Δ*mshA*-complemented strains following INH or ETH treatment, and derivatized to their methyl esters. Analysis by thin-layer chromatography (TLC) allowed for the separation between the short-chain fatty acid (up to 26 carbons in length) methyl esters (FAMEs) and the long-chain mycolic acid methyl esters (MAMEs) (Fig. 6). Treatment of the wild-type *M. tuberculosis* strains and the Δ*mshA*-complemented strains with INH or ETH resulted in inhibition of mycolic acid biosynthesis as shown by the absence of MAMEs on TLC. In contrast, the Δ*mshA* mutants were resistant to mycolic acid inhibition upon treatment with ETH, but not with INH (Fig. 6).

The *mshA* mutants support the premise that ETH inhibits mycolic acid biosynthesis as the *mshA* mutants were resistant to mycolic acid biosynthesis inhibition upon ETH treatment. Previous studies have shown four different mechanisms of ETH resistance in tubercle bacilli, including: (i) target modification (Banerjee *et al*., 1994; Vilcheze *et al*., 2006), (ii) target overexpression (Larsen *et al*., 2002), (iii) intracellular NADH/NAD^+^ ratio alteration (Miesel *et al*., 1998; Vilcheze *et al*., 2005) and (iv) ETH activator inactivation (Baulard *et al*., 2000; DeBarber *et al*., 2000). All four of these phenomena are consistent with ETH being a pro-drug that is activated to form an adduct with NAD and this ETH-NAD adduct inhibits InhA, which results in mycolic acid biosynthesis inhibition (Vilcheze and Jacobs, 2007b; Wang *et al*., 2007).

To address the mechanism by which mutations in *mshA* confer ETH resistance, we can now rule out a number of these known mechanisms. Quantitative PCR analysis demonstrated that the *inhA* mRNA was not upregulated thereby suggesting InhA was not overexpressed. Moreover, we measured NADH/NAD^+^ ratios and found no increase in NADH concentration in the *mshA* mutants. All of these data, coupled with the lack of resistance to INH and the high resistance to ETH, allow us to hypothesize that mycothiol plays a role either in the activation step of ETH or in the formation of the ETH-NAD adduct.

### Mycothiol promotes ETH activation by the *ethA*-encoded mono-oxygenase

As the null mutants showed low (twofold the MIC) to no resistance to INH but showed a high level of resistance to ETH (≥ 6-fold the MIC), we therefore postulated that mycothiol could be involved in either ETH activation or ETH-NAD adduct formation in *M. tuberculosis.* ETH is activated by the NADPH-specific FAD-containing mono-oxygenase EthA (Baulard *et al*., 2000; DeBarber *et al*., 2000; Vannelli *et al*., 2002). We tested the NADPH-dependent mono-oxygenation of ETH by EthA in the presence of mycothiol, and observed an increase in the rate of reaction directly proportional to the increase in mycothiol concentration, suggesting that mycothiol plays a role in the activation steps rather than in the formation of the ETH-NAD adduct (Table 3). Furthermore, replacing mycothiol by a different thiol, such as reduced glutathione, had no effect on the oxidation rate of NADPH (data not shown). This suggests that the increase in EthA activity upon the addition of mycothiol is specific to mycothiol, and does not occur in the presence of another thiol. To test if mycothiol was also required for the formation of the ETH-NAD adduct, the rate of inhibition of InhA by ETH in the presence of NAD^+^, NADPH, EthA and mycothiol was also measured. No formation of the ETH-NAD adduct was observed in these conditions (data not shown), which suggests that mycothiol is not involved in the formation of the ETH-NAD adduct. The mycothiol-dependent increase in the rate of NADPH conversion during the activation of ETH by EthA suggests that mycothiol promotes the activation of ETH by EthA. Two other anti-TB drugs, isoxyl and thiacetazone, are also activated by EthA (Dover *et al*., 2007). We therefore tested if the *mshA* mutants (null and point mutants) were also resistant to isoxyl and thiacetazone and found that they were fully sensitive to both drugs (data not shown). This implies that mycothiol is solely involved in the activation of ETH. We could hypothesize that mycothiol either stabilizes the intermediates formed upon activation of ETH or forms a complex with the active form of ETH, which allows for the formation of the ETH-NAD adduct. More in-depth studies are necessary to fully understand which role mycothiol plays in the activation step.

**Table 3:** The effect of mycothiol on EthA activity.

[Mycothiol] (μM)	[NADPH] s^−1^μM s^−1^	NADPH/EthA mmol s^−1^ (mg protein)^−1^
0	0.0071	1.24 × 10^−4^
4.38	0.0074	1.29 × 10^−4^
17.5	0.0094	1.64 × 10^−4^
26.2	0.0111	1.94 × 10^−4^
43.8	0.0127	2.23 × 10^−4^

### Mycothiol is not required for *M. tuberculosis* growth *in vivo*

**Fig. 7:**
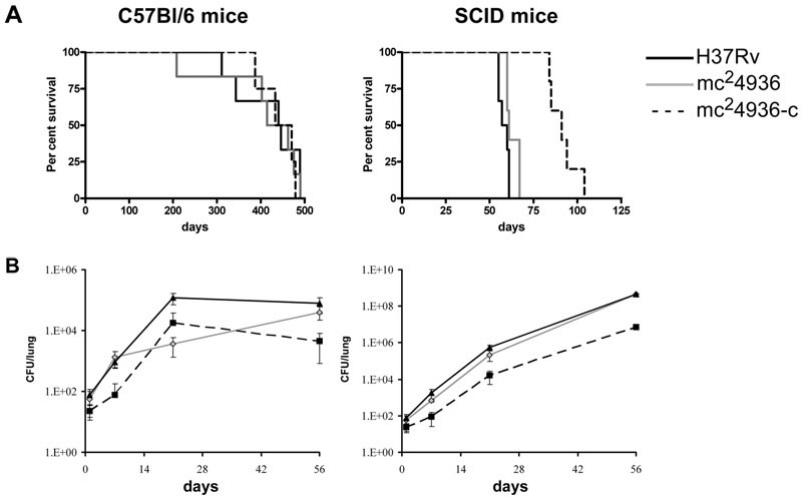
*In vivo* growth of wild-type *M. tuberculosis* H37Rv, mc^2^4936 and its complemented strain mc^2^4936-c following low-dose (≈10^2^ cfu) aerosol infection. A. Survival in immunocompetent C57Bl/6 mice or immunocompromised SCID mice. B. Growth in the lungs of C57Bl/6 mice or SCID mice. c = pMV261::*mshA*.

Mycothiol has been postulated to be essential for *M. tuberculosis* growth *in vivo*. The *mshA* mutant mc^2^4936, which does not synthesize mycothiol, was chosen to study the survival of immunocompetent C57Bl/6 mice and immunocompromised SCID mice following aerosol infection. No significant difference in survival was observed between mice infected with wild-type *M. tuberculosis* and the mycothiol-deficient *mshA* mutant (Fig. 7A). Interestingly, the SCID mice infected with the complemented strain (complementation was done with a replicative plasmid expressing *mshA*) survived 30 days longer than the parent strain. *In vivo* growth of the *mshA* mutant mc^2^4936 in the lungs of immunocompromised and immunocompetent mice was also measured. In SCID mice, the *mshA* mutant and the wild-type *M. tuberculosis* strains grew at the same rate, while the complemented strain grew slightly more slowly, which might explain the differences in survival rates (Fig. 7B). In C57Bl/6 mice, the *mshA* mutant growth was slightly defective after 3 weeks but at week 8 of infection the mycobacterial burden in the lung was comparable between the mutant and the wild-type strain (Fig. 7B).

Our demonstration that *M. tuberculosis*Δ*mshA* strain requires catalase to grow *in vitro*, but not in mice suggests that either the host is not an oxidatively stressed environment or growth in mice induces alternative thiols that may compensate for the absence of mycothiol. Further studies will be required to solve this paradox.

### Concluding remarks

A novel mechanism of ETH resistance has been discovered in *M. tuberculosis* which demonstrates that mycothiol plays a role in the pro-drug activation by the *ethA*-encoded mono-oxygenase. As mono-oxygenases are known to mediate detoxifying reactions, it is reasonable to assume that mycothiol plays a role in other, as yet unidentified, detoxifying reactions. This resistance is a loss of function and is consistent with mycothiol playing a role in the ETH activation process. The requirement for mycothiol in the inhibition of mycolic acid biosynthesis by ETH supports the model that ETH, upon activation with EthA, forms an adduct with NAD, which subsequently inhibits InhA. We hypothesize that novel drugs that bypass this activation step and target InhA directly should be developed as they could lead to the killing of *M. tuberculosis* cells.

Interestingly, the first study by Dubos and Middlebrook (1947) of a medium to grow tubercle bacilli (now referred as Middlebrook 7H9 with OADC supplement) did not add beef liver catalase. A few years later, the discovery by Middlebrook that INH-resistant mutants of *M. tuberculosis* were catalase-negative (Middlebrook, 1954; Middlebrook *et al*., 1954) initiated the need to add catalase to the media to isolate and grow *M. tuberculosis* INH-resistant strains. Our study shows that there exists at least one class of INH- or ETH-resistant *M. tuberculosis* mutants that would not readily grow on media without catalase and may provide an explanation why certain clinical isolates are difficult to grow. Furthermore, the finding that three *mshA* mutants, when complemented with a wild-type copy of *mshA*, still possessed a low-level resistance to INH but were fully sensitive to ETH suggests that these mutants must have acquired a secondary mutation. Therefore, there may exist additional mechanisms of INH resistance yet to be discovered involving other redox pathways.

## Experimental procedures

### Bacterial strains, plasmids, phages and media

The *M. tuberculosis* strains (H37Rv, CDC1551 and Erdman) were obtained from laboratory stocks. The strains were grown in Middlebrook 7H9 medium (Difco) supplemented with 10% (v/v) OADC enrichment (Difco), 0.2% (v/v) glycerol and 0.05% (v/v) tyloxapol. The solid medium used was the same as described above, with the addition of 1.5% (w/v) agar. The plasmids pMV261 and pMV361 were obtained from laboratory stocks. Hygromycin was used at concentrations of 50 mg l^−1^ for mycobacteria and 150 mg l^−1^ for *Escherichia coli.* Kanamycin was used at concentrations of 20 mg l^−1^ for mycobacteria and 40 mg l^−1^ for *E. coli.*

### Isolation of INH- and ETH-resistant spontaneous mutants

*Mycobacterium tuberculosis* H37Rv mutants were isolated from non-mutagenized cultures grown in the media described above. The cultures were incubated by shaking at 37°C to late log phase. Ten-fold serial dilutions were then plated on agar plates (media described above) containing INH (0.2 μg ml^−1^) and ETH (5 or 10 μg ml^−1^). The plates were then incubated at 37°C for 6 weeks.

### MIC determination

The strains were grown to an OD_600_≈ 1.0. Ten-fold serial dilutions were plated on plates containing INH (0, 0.1, 0.2, 0.25, 0.3, 0.4, 0.5, 0.6, 0.8, 1 μg ml^−1^) or ETH (0, 2.5, 5, 10, 15, 20 μg ml^−1^). The MIC was determined as the concentration of drug that reduced the number of colony-forming units (cfu) ml^−1^ by 99%. MICs were also determined using the MTT assay (Martin *et al*., 2005).

### Determination of NADH and NAD^*+*^ cellular concentrations

*Mycobacterium tuberculosis* strains were grown to log phase. The cultures (12 ml) were spun, and the cell pellets were re-suspended in 0.2 M HCl (1 ml, NAD^+^ extraction) or 0.2 M NaOH (1 ml, NADH extraction). After 10 min at 55°C, the suspensions were cooled to 0°C, and neutralized by adding 0.1 M NaOH (1 ml, NAD^+^ extraction) or 0.1 M HCl (1 ml, NADH extraction). After centrifugation, the supernatants were collected, filter-sterilized and frozen. The concentration of NAD^+^ (or NADH) was obtained by measuring spectrophotometrically the rate of 3-[4,5-dimethylthiazol-2-yl]-2,5-diphenyltetrazolium bromide reduction by the yeast type II alcohol dehydrogenase in the presence of phenazine ethosulphate at 570 nm, which is proportional to the concentration of nucleotide (Leonardo *et al*., 1996; San *et al*., 2002).

### Quantification of the *inhA* expression levels

Total RNA extractions, cDNA synthesis and quantitative PCR with molecular beacons were performed in triplicate, as described previously (Larsen *et al*., 2002). *inhA* levels were normalized to *sigA* expression.

### Quantification of mycothiol contents

The *M. tuberculosis* strains (45 ml) were grown to stationary phase for 2 weeks. Samples (9 ml) were transferred into conical tubes and centrifuged. The cell pellets were re-suspended in either 0.5 ml of mBBr reagent (20 mM HEPES pH 8 + 2 mM monobromobimane in acetonitrile/water 1/1, v/v) or 0.5 ml of NEM reagent (20 mM HEPES pH 8 + 5 mM *N*-ethylmaleimide in acetonitrile/water 1/1, v/v)). The suspensions were heated at 60°C for 15 min and spun down. The supernatants (0.5 ml) were treated with 5 M methane sulphonic acid (2 μl) and frozen. The samples were subjected to HPLC analysis as described earlier (Newton *et al*., 2000b).

### Quantification of the total thiol concentration

*Mycobacterium tuberculosis* strains were grown to stationary phase and spun down. The cell pellets were washed with PBS and then re-suspended in 1 ml of PBS. Glass beads were added (0.2 ml), the suspensions were lysed using the Thermo Scientific FastPrep machine (45 s, speed 6, three times) and spun down, and the supernatants were filter-sterilized. The total thiol concentration was obtained using Ellman's reagent by measuring spectrophotometrically, at 412 nm, a 1 ml solution containing 50 mM Tris (pH 8.0), 5 mM 5,5′-dithiobis(2-nitrobenzoic acid) (10 μl), and the lysate to quantify (ε_412 nm_ 2-nitro-5-thiobenzoate anion is 14 150 M^−1^ cm^−1^).

### Construction of the *ΔmshA* strains

*Mycobacterium tuberculosis mshA* was replaced by a hygromycin cassette using the specialized transduction system previously described (Bardarov *et al*., 2002). Briefly, a 1 kb region flanking the left and right sides of *mshA* was PCR-amplified from *M. tuberculosis* genomic DNA using the following primers (the cloning sites are underlined):

LL TTTTTTTTCCATAAATTGGGGGCCGCGCTGACCTCACTG,
LR TTTTTTTTCCATTTCTTGGGACGGCGCTGGGCGATCAAC,
RL TTTTTTTTCCATAGATTGGCCTGGTAGCGGTGGGCAAGC,
RR TTTTTTTTCCATCTTTTGGGCGGGCCGATCGCGACCTTG.

The PCR fragments were cut with Van91I and cloned into p004S. The resulting cosmid was sequenced before digesting with PacI. The linearized cosmid was ligated to the PacI-cut shuttle phasmid phAE159 and the resulting phasmid, phAE222, was packaged *in vitro* (Gigapack II, Stratagene). High-titre phage lysates were used to transduce *M. tuberculosis* H37Rv, CDC1551 and Erdman as described previously (Vilcheze *et al*., 2006). The plates were incubated at 37°C for 8 weeks. The transductants were checked for the deletion of *mshA* by Southern analysis (the genomic DNA of the transductants was cut with BglII and probed with the right flank of *mshA*) (Fig. S1).

### Complementation of the *mshA* mutants

The wild-type *M. tuberculosis mshA* gene was amplified from *M. tuberculosis* chromosomal DNA using the following primers: mshAF CGGCAGCTGTTCGGTTCCTGCAAGGATGG (PvuII site underlined), mshAR GCGGAATTCTCGGCAAGGAGGAAGTCACG (EcoRI site underlined). The PCR product was digested with PvuII and EcoRI and ligated to the replicative *E. coli* mycobacterial shuttle vector pMV261 (Stover *et al*., 1991) (http://www.aecom.yu.edu/tbresearch/Resources/Vectors/261.html) restricted by PvuII and EcoRI or the integrative *E. coli* mycobacterial shuttle vector pMV361 (http://www.aecom.yu.edu/tbresearch/Resources/Vectors/361.html) (Stover *et al*., 1991) restricted by PvuII and EcoRI. The *mshA* mutant strains were then transformed with the plasmid pMV361::*mshA* or pMV261::*mshA*, using the following protocol. The strains (20 ml of cultures) were grown at 37°C to an OD_600_≈ 0.8, washed twice with a 10% aqueous glycerol solution and re-suspended in 0.4 ml of a 10% aqueous glycerol solution. The cell suspensions (0.175 ml) were added to the plasmid (2 μl) and electroporated (2.5 kV, 25 μFd, 1000 Ω). Medium (1 ml) was added, and the suspension was incubated at 37°C for 24 h and plated on Middlebrook plates containing kanamycin (20 mg l^−1^). The plates were incubated at 37°C for 6 weeks.

### Analysis of FAMEs and MAMEs

*Mycobacterium tuberculosis* strains were grown to log phase, diluted to an OD_600_≈ 0.3, treated with INH (0.5 mg l^−1^) or ETH (15 mg l^−1^) or no drug for 4 h, and then labelled with [1-^14^C]-acetate (10 μCi) for 20 h at 37°C. The cultures were spun down and washed once with water. The cell pellets were saponified, methylated, extracted and analysed by TLC using hexane/ethyl acetate 95/5 as the elution system (three elutions were performed) (Vilcheze and Jacobs, 2007a).

### EthA enzymatic activity assay

The his-tagged EthA was produced, as previously described (Dover *et al*., 2007). The activity of EthA was determined by monitoring the absorbance decrease of NADPH at 340 nm (ε_340 nm_ = 6.22 mM^−1^ cm^−1^). All the reactions were catalysed by ∼1 μM EthA and performed in 50 mM Tris/HCl, pH 7.5. Double reciprocal plots were used to determine the *k*_cat_ of the oxidation of NADPH. For measuring the effect of mycothiol,reaction mixtures contained 200 μM NADPH and varying mycothiol concentrations.

### Mice experiments

SCID mice and C57Bl/6 mice (Jackson Laboratories) were infected via the aerosol route using a 10^6^ cfu ml^−1^ mycobacterial suspension in PBS containing 0.05% Tween 80 and 0.04% antifoam. Three mice from each group were sacrificed after 24 h of infection and lung homogenates were plated on Middlebrook 7H10 plates containing the appropriate antibiotic to determine the initial infection dose. At 1, 3 and 8 weeks post infection, three mice were sacrificed to determine the bacterial burden in the lung, spleen and liver. Five mice were left for survival experiments.

## Supplementary material

This material is available as part of the online article from: http://www.blackwell-synergy.com/doi/abs/10.1111/j.1365-2958.2008.06365.x

(This link will take you to the article abstract).
