# Supplementary material for: Mycothiol biosynthesis is essential for ethionamide susceptibility in Mycobacterium tuberculosis
Source: Mol Microbiol. 2008 Jul 21;69(5):1316–29. doi: 10.1111/j.1365-2958.2008.06365.x (PMC2628429; doi:10.1111/j.1365-2958.2008.06365.x)
Supplement: Supplementary file 1 [file mmi0069-1316-SD1.pdf]

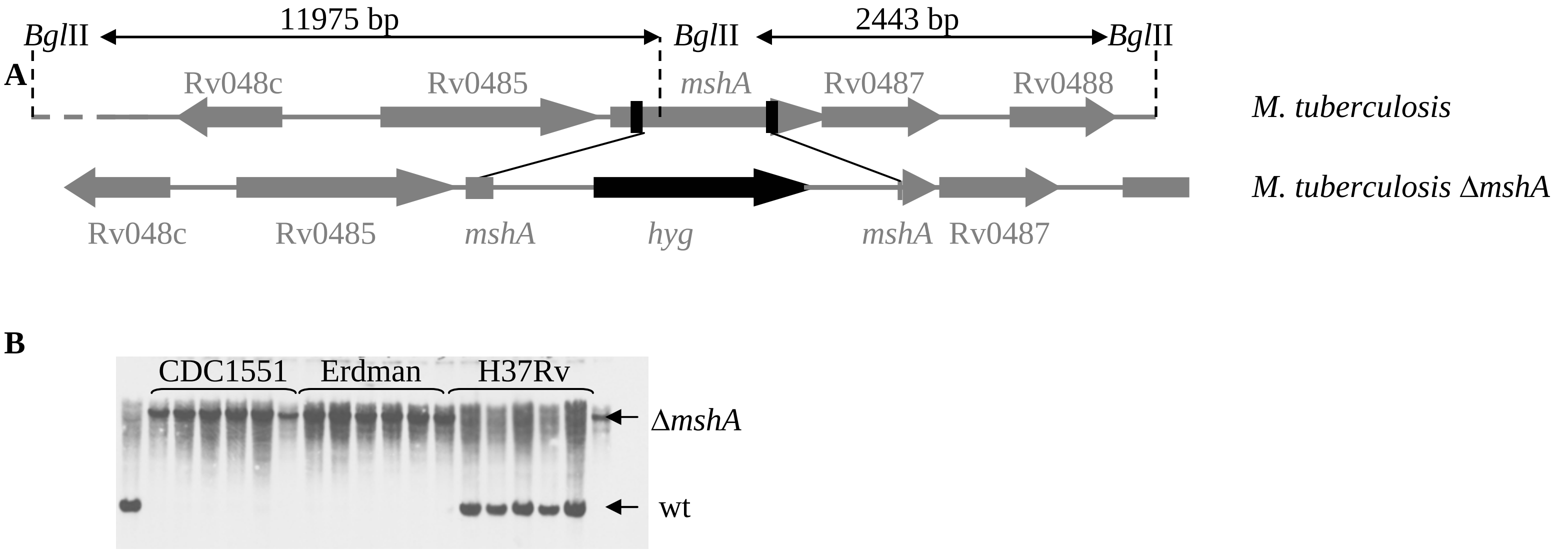

Fig. S1: *mshA* deletion in *M. tuberculosis*. A. The *mshA* gene of three *M. tuberculosis* strains was replaced by a hygromycin cassette using specialized transduction. B. Southern analysis of wild-type and Δ*mshA* genomic DNA after digestion with *Bgl*II (expected size for wt 2443 bp and for Δ*mshA* 14418 bp).
